# Hypertension and human immunodeficiency virus: A paradigm for epithelial sodium channels?

**DOI:** 10.3389/fcvm.2022.968184

**Published:** 2022-08-25

**Authors:** Katongo H. Mutengo, Sepiso K. Masenga, Naome Mwesigwa, Kaushik P. Patel, Annet Kirabo

**Affiliations:** ^1^School of Medicine and Health Sciences, HAND Research Group, Mulungushi University, Livingstone Campus, Livingstone, Zambia; ^2^School of Public Health and Medicine, University of Zambia, Lusaka, Zambia; ^3^Department of Medicine and Dentistry, Kampala International University, Kampala, Uganda; ^4^Department of Cellular and Integrative Physiology, University of Nebraska Medical Center, Omaha, NE, United States; ^5^Department of Medicine, Vanderbilt University Medical Center, Nashville, TN, United States

**Keywords:** hypertension, inflammation, HIV, cardiovasclar disease, ENaC (epithelial Na+ channel)

## Abstract

Hypertension is a risk factor for end organ damage and death and is more common in persons with HIV compared to the general population. Several mechanisms have been studied in the pathogenesis of hypertension. Current evidence suggests that the epithelial sodium channel (ENaC) plays a key role in regulating blood pressure through the transport of sodium and water across membranes in the kidney tubules, resulting in retention of sodium and water and an altered fluid balance. However, there is scarcity of information that elucidates the role of ENaC in HIV as it relates to increasing the risk for development or pathogenesis of hypertension. This review summarized the evidence to date implicating a potential role for altered ENaC activity in contributing to hypertension in patients with HIV.

## Background

The epithelial sodium channel (ENaC) is a membrane-bound ion channel that transports sodium [Na^+^] ions and is abundantly expressed in various epithelial cells (1, 2). It plays a critical role in blood pressure and volume control, classically through its regulation of sodium reabsorption in the distal nephron (3). It is known that the role of ENaC in blood pressure regulation is mainly controlled by the activation of the renin-angiotensin-aldosterone system (4–6). However, other extrarenal factors have also been implicated in ENaC activation including the reactive oxygen species (ROS) (6, 7).

ROS are produced as a by-product of normal aerobic metabolism, and levels are regulated by critical antioxidant enzymes, which include superoxide dismutase, the glutathione peroxidases, catalase, and glucose-6-phosphate dehydrogenase (8). Under regulated conditions, the ROS are essential for immune signaling pathways and are incorporated into immune responses (9, 10). However, excess ROS are detrimental to vascular health and are produced when there is increased activity of reduced nicotinamide adenine dinucleotide phosphate (NADPH) oxidase, the main enzyme responsible for generating superoxide from oxygen (8, 11). Other ROS-generating enzymes include xanthine oxidases, and cyclooxygenases (12). Importantly, both human immunodeficiency virus (HIV) and hypertension are associated with an increase in ROS generation (13–15), and therefore potentially contribute to the upregulation of ENaC. While clear and well-documented evidence supports the role of ENaC in the pathogenesis of salt-sensitive hypertension (6, 16, 17), it is not clear if there is a potential synergism between HIV and ENaC, despite recent evidence from our research indicating a high prevalence of salt-sensitivity in people with HIV (PWH) who had hypertension (18). Further studies to elucidate the link between HIV, salt sensitivity and the additive role that ENaC may play remains to be explored.

The major risk factor for cardiovascular diseases (CVDs) is hypertension and sub-Sahara Africa has a weighty synchronization of hypertension and HIV (19–22), making it a prime site for this area of research. Easy access to combined antiretroviral therapy (cART) as well as newer innovations in HIV programs has seen HIV transition from a once fatal disease to a chronic and manageable condition. Even regions hardest hit by the epidemic such as Eastern and Southern Africa; which account for 54% of the global HIV burden, have observed a decline in new HIV infections by 28% and deaths by 44% between 2010 and 2018 (23). However, these positive strides have also seen a progression of non-infectious chronic complications such as CVDs (24–26). HIV has been known to confer about 1.5 to 2-fold increased relative risk to CVDs such as myocardial infarction, heart failure and stroke (24, 25, 27, 28), with hypertension being a consistent underlying risk factor. The association between HIV-related factors and traditional risk factors for atherosclerosis, also associated with hypertension, has been implicated as causing a higher risk of CVDs in PWH (29–32). Therefore, major research efforts need to be made to cope with this rising burden of CVDs. This review provides evidence linking a possible role for activation of ENaC during HIV infection in contributing to hypertension.

## The epithelial sodium channel

**FIGURE 1:**
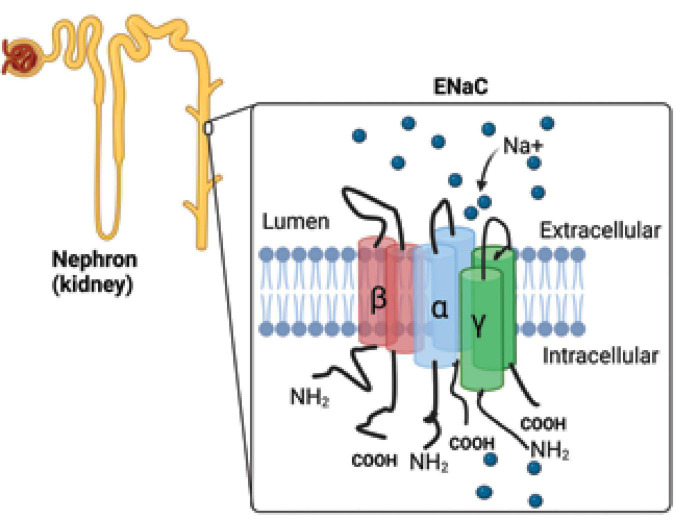
Epithelial sodium channel. The epithelial Na+ channel (ENaC) is responsible for the reabsorption of sodium ions by the epithelia lining the distal part of the kidney tubule (collecting duct). The ENaC consists of three main subunits called α, β, and γ. NH_2_, amino terminal group; COOH, carboxyl group, Na+, sodium ions.

The epithelial Na^+^ channel (ENaC; Figure 1) is a membrane-bound ion channel that is selectively permeable to Na^+^ ions and consists of three main subunits called α, β, and γ with a fourth (δ) that is analogous in function to the α subunit found in some tissues where it acts in place of α (1, 2, 33, 34).

There are four major ENaC regulatory mechanisms: The first is Na^+^ self-inhibition in which Na^+^ can either induce an allosteric change in ENaC or slowly inhibit the channel over time (35–37). This type of regulation enables Na^+^ reabsorption to be controlled via concentration of Na^+^ in the distal nephron. ENaC regulation also occurs via post-translational proteolytic cleavage, resulting in the abandonment of inhibitory pathways, making the channel more likely to remain open (38–40). Lipid signaling molecules also play a role in both increasing and decreasing the opening probability of ENaC via both binding to cationic sequences and inhibitory lipids (41, 42). Finally, aldosterone may also help regulate ENaC regulation in several different ways. Angiotensin II (Ang) II also influences this process by promoting secretion of the key ENaC regulator, aldosterone from the adrenal zona glomerulosa (4). The activation and expression of serum-and-glucocorticoid-induced kinase (SGK1) through the influence of aldosterone contributes to the increased expression and opening probability of ENaC at the plasma membrane (43–45).

ENaC’s role in the kidney, where it acts as a control mechanism for Na+ and K^+^, has been extensively studied (5, 6, 39, 45, 46). It is typically expressed in the aldosterone-sensitive distal nephron (ASDN) of epithelial cells. In Na^+^ control, it acts as a rate-limiting step for transepithelial Na^+^ reabsorption, total body net Na^+^ content, fluid volume, and blood pressure in the nephron collecting duct (47). It is also critical to the secretion of K^+^ in ASDN (48). Elevation of serum potassium is considered to be one of the most important agonist for aldosterone secretion (49). Aldosterone, as aforementioned, acts through the mineralocorticoid receptor to modulate changes in blood volume and sodium balance by increasing ENaC abundance and activity (4, 50). Opening of the ENaC channels results in a lumen to intracellular Na+ flux, which is facilitated by the negative electrical potential and the steep Na^+^ concentration gradient across the apical membrane. Na+ is then actively transported across the basolateral membrane by the Na+/K + ATPase in exchange for potassium, and potassium then exits the apical membrane K+ channels (51). Hyperkalemia and hypokalemia are common in HIV, although their impact on renal salt handling and ENaC remains unexplored. For example, commonly used drugs such as Amphotericin B to treat cryptococcal meningitis in HIV, chronic diarrhea, AIDS-associated enteropathies as well as detectable HIV mRNA levels were associated with hypokalemia (52–54). Conversely, hyperkalemia has been seen with the use of high-dose trimethoprim-sulfamethoxazole or intravenous pentamidine for pneumocystis jirovecii pneumonia attributed to an amiloride-like mechanism by blocking apical membrane sodium channels in the mammalian distal nephron (55–57). On the other hand, a study exploring the spectrum of electrolyte abnormalities in black African people living with human immunodeficiency virus and diabetes mellitus (DM) found that comorbidity of HIV and DM increased the chance of hypokalemia by 97% for every unit increase in DM duration attributed to a greater degree of insulin resistance, as both conditions progress (58). On the contrary, the non-HIV infected in this study had a 10% increased chance of hyperkalemia for every unit increase in DM attributed to dysautonomia in long- standing DM which predisposes to hyporeninaemic hypo-aldosteronism and associated hyperkalaemia (58). *In vitro* data suggest that individuals infected with HIV have alterations in transcellular K+ transport. Caramelo et al. (59) demonstrated that L-arginine–induced, rapid-onset hyperkalemia, was only present in the HIV infected and not the healthy controls suggesting that HIV-positive individuals have a systemic derangement in transmembrane K+ transport that is coincident with inhibited aldosterone response. Although inhibition of aldosterone has traditionally been thought to downregulate ENaC function, it has been postulated that ENaC activity is high in some instances when there are no significant changes in aldosterone, termed aldosterone-independent ENaC activation, where Vasopressin is implicated in the increased activity (60). However, it remains unclear whether disruption of transmembrane K+ transport with inhibited aldosterone response found in HIV (59) can affect ENaC function and activation.

In addition, extrarenal factors such as immune activation through the generation of ROS also upregulate the ENaC (7). ENaCs have also been found to be expressed in immune cells such as the dendric cells (DCs), which contribute to the pathogenesis of salt-sensitive hypertension (61, 62). It is worth noting that DCs are also the main site of HIV seeding, altering their function (63, 64). Whether this contributes to the upregulation of ENaC in these cells remains to be determined.

### Epithelial sodium channel, salt-sensitive hypertension and immune activation

While studies on the impact of blood pressure changes and ENaC function in HIV are lacking, PWH have been widely reported to have a higher prevalence of hypertension than the general population (65, 66). A study by Masenga et al. (18) showed that PWH who were hypertensive, also demonstrated a high salt sensitivity compared to those who were normotensive and HIV-negative individuals. Because hypertension is driven by dysregulated stimulation of the RAAS (46, 67, 68), the process that also upregulates ENaC function (4–6), an increase in blood pressure can be viewed as a driver or a consequence of increased ENaC activity. Genetic variations in blood pressure responses have been extensively studied in the general population, revealing that individuals with rare variants of RAAS pathway were 1.55 times more likely to have salt-sensitivity compared to non-carriers, suggesting an association between RAAS and Na+ transporters (69). Although the effects of HIV and cART contribute to oxidative stress which contributes to RAAS activation (9, 70–73), investigating whether specific genetic variations contribute to the upregulation of ENaC in PWH is warrantable.

Mutations in ENaC result in human hypertension or hypotension, connecting it to the regulation of blood pressure (74). Beyond its role in the kidney, ENaC has been implicated in the interplay between hypertension, salt sensitivity, and the immune system (17, 61). The immune system’s response to hypertension may be a function of self-tolerance loss. In this case, hypertension would be considered an autoimmune disease. There is also evidence to suggest that the immune system plays an important role in salt sensitivity of blood pressure (SSBP). SSBP is defined as changes in blood pressure (BP) which mirror changes in dietary salt intake/depletion (75). The immune system’s link to hypertension and salt-sensitivity is based on the activity of antigen-presenting cells (APCs), particularly macrophages, DCs, and B cells. In response to hypertensive stimuli, such as norepinephrine and dietary salt, these APCs enter the vasculature and kidney along with T lymphocytes where they converge in aiding Na^+^ retention and vasoconstriction leading to the elevation of blood pressure and consequent end-organ damage (44, 62, 76–78). Coincidentally, APCs like monocytes stand out as the most abundant immune cell type found in atherosclerotic plaques (79, 80). There are three distinct types of human monocytes based on their expression of cluster of differentiation-14 (CD14) and CD16 soluble surface markers: classical with little or no CD16 (CD14++CD16-), intermediate with expression of both CD14 and CD16 (CD14++CD16 +), and non-classical with low CD14 (CD14*^low^*CD16++) monocytes (79, 81). The classical monocytes are rapidly recruited to invade the inflamed tissue during injury or inflammation and contribute to immunological responses. Such a response comprises of recognizing and removing microorganisms and dying cells while non-classical monocytes patrol the endothelium as part of innate surveillance (82). The intermediate monocytes are recruited at a later stage of inflammation and involved in pro-inflammatory response (82). Interestingly high levels of circulating monocytes with CD16 and CD14 have been observed during HIV-infection (83–85). These immune cells are key drivers of atherosclerotic processes leading to CVDs (85–88). In fact, soluble CD14 has been found to be elevated in PWH with hypertension (89), although the contribution of ENaC to these pathoimmune responses in PWH remain to be studied. Furthermore, Loperena et al. (90) observed a direct relationship between 48-h uniaxial hypertensive stretching of endothelial cells and the transformation of classical monocytes (CD14++CD16-) into intermediate monocytes (CD14^++^CD16^+^). Loperana et al. further demonstrated that these CD14++CD16+ monocytes produced significant pro-inflammatory cytokines (interlukin-6 [IL-6], interlukin-1β [IL-1β], interlukin-23 [IL-23], and tumor necrosis factor α [TNF-α]) that drove T-cell proliferation in the human volunteers. This study excluded PWH and was therefore unable to make comparisons in this group, but a hypothesis can be postulated on the basis of a loose association with other studies showing an elevation of the monocyte-soluble marker CD14 in hypertensive PWH (89) and with upregulation of ENaC in DCs, which are simply activated monocytes (61), and increased CD14^++^CD16^+^ markers with high salt concentration (91).

Additionally, recent studies have identified a connection between EnaC, hypertension, and innate immunity. An exploration of Na^+^-dependent EnaC activity in DCs and its signaling mechanisms has shown that, as Na^+^ concentration increases extracellularly, both NADPH-oxidase activation and superoxide generation also increase (91). This leads to isolevuglandins (IsoLG)-adduct proteins, which are formed from lipid peroxidation of arachidonic acid and are extremely immunoreactive. IsoLG-adduct proteins produced in monocyte-derived DCs can lead to immune tolerance depletion in salt-induced hypertension (62, 92). As IsoLG-adduct proteins increase in an EnaC-dependent mechanism through elevated extracellular high salt conditions, the surface expression of CD83 and CD16, and the B7 ligands CD80 and CD86, increases in a process important for the maturation of DCs and antigen presentation (91, 92). On the other hand, contrasting effects of HIV-1 on DC phenotype and/or function have been reported. While some studies have reported an upregulation of the markers CD80, CD83, and CD86 upon exposure to HIV-1 or its proteins (93), others reported no effect (94), creating a controversy as to whether EnaC activation can also be variable, as the aforementioned markers are also associated with EnaC activation.

**FIGURE 2:**
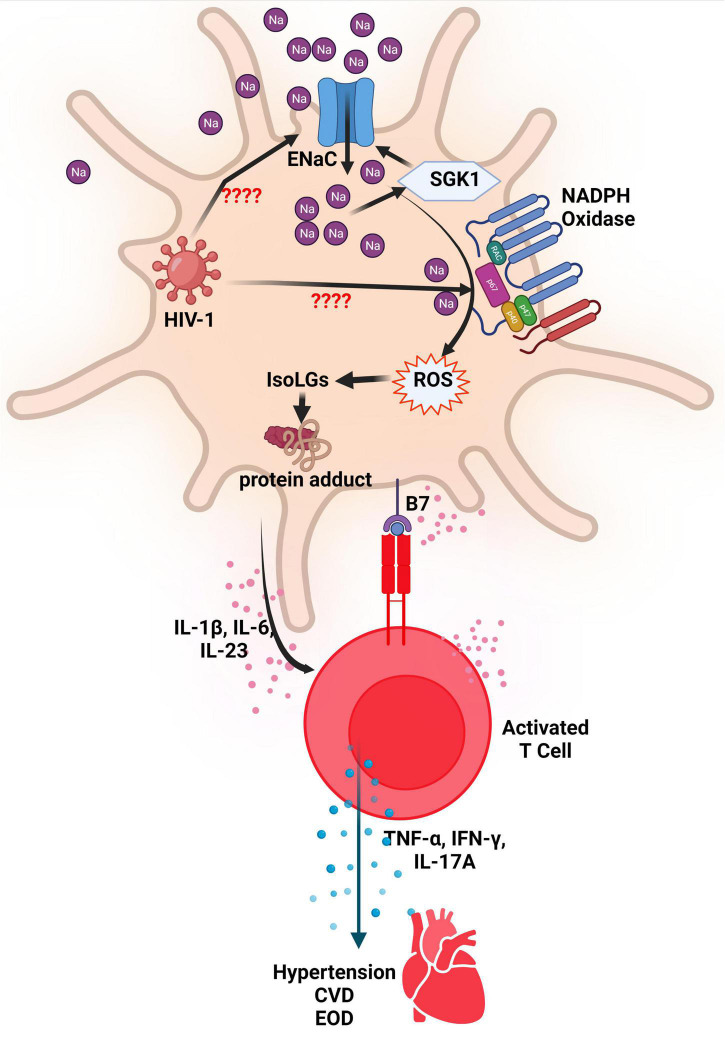
ENaC and NADPH oxidase activation in DCs resulting in hypertension. The increased activity of the endothelial sodium channel (ENaC) leads to an increase in Na+ ions that activates the enzyme NADPH Oxidase. The increased activity of NADPH Oxidase results in production of reactive oxygen species (ROS) which leads to formation of Isolevuglandins (IsoLGs). IsoLGs adduct to intracellular proteins and become neoantigens that are presented to T cells to activate them. Activated T cells produce inflammatory cytokines that contribute to development or progression of hypertension, cardiovascular disease (CVD) and end organ damage (EOD). It is thought that HIV-1 activates ENaC and NADPH in antigen presenting cells resulting in similar effects. SGK1, serum-and-glucocorticoid-induced kinase; NADPH, Reduced nicotinamide adenine dinucleotide phosphate; Na, sodium; HIV-1, human immunodeficiency virus type 1; IL, interleukin; TNF-α, tumor necrosis factor alpha; IFN-γ, interferon gamma; CVD, cardiovascular disease; EOD, end organ damage.

SGK-1, an intracellular salt-sensing kinase, plays a role in blood pressure modulation and EnaC regulation in the distal convoluted tubule (61). In APCs, SGK-1 controls the increased expression of the α- and γ-subunits of EnaC as mediation in salt-sensitive hypertension. This regulation results in the production of IsoLG-adducts and interleukin-1β (IL-1β) as well as the activation of T cells (61). SGK1 activity increases autoimmunity as it restricts the function of Treg cells and reciprocally regulates the development of T helper 17/regulatory T cell (Th17/Treg) balance. More Th17 means more IL-17 and therefore contribute to the development of hypertension (95, 96). There is also evidence of a possible connection between IL-1β and salt-sensitive hypertension (97, 98). According to both animal and human studies, cytokines like IL-1β generate a pro-inflammatory state which may result in heightened blood pressure due to variation in renal, endothelial, and immune responses. In mice specifically, the pharmacological inhibition of IL-1β, targeted antibody treatment of IL-1β, and genetic deletion have all proven to lead to decreased blood pressure (99–101). However, it remains unclear whether the pro-inflammatory state induced by IL-1β is necessarily a good indicator of resultant hypertension in PWH, especially since HIV-1 infection in human monocytes efficiently induce IL-1β expression and inflammasome activation (102). This inflammation is also exacerbated by high salt concentration which has been shown to aid in the production of IL-1β via blood mononuclear cells and the EnaC-dependent production of IL-1β in DCs (91, 103). A summary of the possible interaction between HIV, EnaC, ROS and activated T cells in the evolution of hypertension are presented in Figure 2.

## Epithelial sodium channel and cardiovascular disease

Evidence now strongly suggests that vascular EnaC contributes to the pathogenesis of arterial hypertension propagated by endothelial dysfunction (104). Vascular endothelium is a single layer of cells that forms the first barrier in the blood vessel lumen. In larger vessels, this layer is in contact with vascular smooth muscle, collagen and elastic fibers (105). The endothelium is a biologically active cell layer that produces mechanical sensors and transducers which maintain vascular homeostasis, vascular tone, and vascular integrity (105, 106). Normal endothelial function requires the regulated balance in these mechano-sensors and transducers. This balance is a function of a number of endothelium-derived relaxation factors such as vasodilator prostaglandins, nitric oxide (NO), endothelium-dependent hyperpolarizing factors, and endothelium-derived contraction factors (107–109). However, NO appears to be the main contributor to vascular homeostasis (110, 111).

There is an inverse relationship between NO availability and the generation of ROS. Excess ROS is detrimental to vascular endothelium and production is increased in situations of reduced NO, the major factor in vascular homeostasis (110–113). The reduction in the production of NO occurs mainly through two processes. The first is the increased activity of NAD(P)H oxidase, an enzyme responsible for the generating superoxide from oxygen. The superoxide reacts with NO to form peroxynitrite (OONO-), thereby oxidatively inactivating the NO (11). Second is the uncoupling of the oxidoreductase enzyme endothelial NO synthase (eNOS). This means that eNOS is converted from an enzyme that oxidizes L-arginine to NO into one that reduces molecular oxygen to a superoxide anion which is one of the main ROS (11, 114). The increase in these endothelial ROS induces lipid oxidation, leading to the production of oxidized low-density lipoprotein (LDL) cholesterol (115, 116). Oxidized LDL is further internalized in the endothelium by binding to a transmembrane glycoprotein, lectin-like oxidized low-density lipoprotein (LDL) receptor-1 (LOX-1) (117). Excess sub-endothelial deposition of oxidized LDL activates the endothelium to increase expression of cellular adhesion molecules that stimulate platelet aggregation, increased attachment and migration of inflammatory cells into the intima, such as monocytes which transform into lipid-laden macrophages called foam cells (106, 117, 118). There is also increased production of vasoconstrictors and proliferation and migration of vascular smooth muscle cells and deposition of collagen (117). ROS also induce direct toxicity to endothelial cells leading to apoptosis and inflammation further repeating the vicious cycle of the ROS generation (106). Eventually, these processes cause stiffness and narrowing of blood vessels; an atherosclerotic process which leads to hypertension. The common traditional risk factors for CVDs such as obesity, hypertension, diabetes mellitus, smoking, aging, and hypercholesterolemia can further increase the possibility of the generation of ROS (119–122). These risk factors are heightened in HIV-infection and trigger several pathological pathways that cause endothelial dysfunction and subsequent atherosclerosis (14, 70, 71). ROS also stimulate EnaC activity (6, 7), and possibly can play an additive or multiplicative role in the onset of hypertension in PWH.

A study by Jia et al. found that EnaC activation mediates endothelial permeability, which in turn promotes macrophage infiltration, M1 polarization, and oxidative stress leading to cardiac fibrosis and diastolic dysfunction in female mice with dietary obesity (44). In human studies, there is increased expression of the EnaC in neutrophils from hypertensive patients suggesting an inflammatory role (123). Upregulation of the EnaC has been linked to stiffening of the cortical actin cytoskeleton in endothelial cells including those in the coronary vessels, impaired endothelial NO release, increased oxidative stress, increased vascular permeability, and immune activation (124). Incidentally, similar mechanisms are induced by HIV (14, 15, 28, 125), further addressing the possibility of why PWH are at a higher risk of developing CVDs. These factors can lead to modulation of vascular tone and stimulation of tissue remodeling, leading to heart failure with preserved systolic function (HfpEF) (124).

While the cause of hypertension in HIV is multifaceted, it can generally be accepted that ROS play a crucial role in the onset and sustenance of hypertension. Reduction in NO release is the hallmark of endothelial dysfunction which triggers a cascade of events leading to hypertension and this reduction is enhanced by high Na+ concentration (126). A study on the effects of HIV on the pulmonary vasculature showed that HIV causes endothelial damage and mediator-related vasoconstriction through stimulation by the envelope *gp 120*, including direct release and effects of endothelin-1 (vasoconstrictor), interlukin-6 and TNF-α (127). The inflammatory mediators, IL-6 and TNF-α, which induce mediator-related vasoconstriction, also contribute to formation of ROS, which are important for the upregulation of ENaC (6, 7, 128). Upregulation of ENaC further leads to a cycle of impaired endothelial NO release, increased oxidative stress, increased vascular permeability, and immune activation which contribute to atherosclerosis and hypertension (124, 128). Increasing NO bioavailibity is important for smooth muscle relaxation and overall vascular health (110, 111). However, oxidative processes reduces NO bioavailability (11, 129), and these can be induced by both HIV and ENaC upregulation. HIV therefore likely plays a synergistic role in the development of hypertension through its possible indirect role in the vascular ENaC activation.

Non-invasive measurements such as flow-mediated dilatation (FMD) of the brachial artery have been used as surrogates to determine endothelium-dependent vasorelaxation (129). A study by Oliviero et al. showed that FMD is impaired in naive untreated PWH versus healthy participants (130), indicating inadequate release of vasodilators, including nitric oxide, which cause dilation in a healthy artery (131). Oliviero et al. further found a strong inverse correlation between FMD values and HIV mRNA levels which favored the direct role for early stage of viral infection in vascular dysfunction independent of cART and metabolic factors. On the contrary, another study looking at FMD reported no significant changes in the HIV-treatment naïve, HIV on cART and healthy participants, despite high levels of inflammatory markers in the HIV-treatment naïve (132). Although generally, cART has been linked to the development atherosclerotic changes mostly contributed by ROS generated as a result of lipid abnormalities (72, 133–136). With scarcity of data linking the ENaC to cardiac pathologies and HIV, more studies are warranted to examine this connection.

### HIV-1 encoded proteins and inflammation

Glycoprotein 120 (gp120) is a critical protein in the binding of the HIV to cell surface receptors of target cell. It is expressed on the outer layer on the virus, in free form in the body fluids of infected people, as well as the virus infected cells (137). Gp120 is critical for virus infection, as the protein is necessary for binding to specific cell surface receptor CD4, mainly expressed on CD4+ T cells, and the coreceptor [e.g., chemokine receptor C-C Motif Chemokine Receptor 5 (CCR5) or C-X-C chemokine receptor type 4 (CXCR4)] (138). The molecular and cellular mechanisms through which gp120 induce vascular injury is multifaceted. Studies have demonstrated that gp120 acts synergistically with HIV nuclear protein trans-activator of transcription (Tat) to stimulate release of endothelial microvesicles which further confer pathological effects on endothelial cells by inducing inflammation, oxidative stress, and senescence as well as enhancing susceptibility to apoptosis (15). This HIV glycoprotein also acts as an agonist to proinflammatory cytokines such as TNF- α (139), endothelial monocyte-activating polypeptide II (140) and interleukin-6 and interleukin-8 (15), as well as induce apoptotic signaling transduction pathways through the p38 mitogen-activated protein kinase (MAPK) (141). All these pathways contribute to ROS generation, endothelial apoptosis, platelet aggregation and formation of atheroma and are also known to affect ENaC and thus may interact during HIV to develop hypertension (6, 7).

On the other hand, negative regulatory factor (Nef) is the most frequently transcribed HIV accessory protein in the early stages of viral infection. It is critical for maintaining virus production and progression to acquired immune deficiency syndrome (AIDS) (142). Nef has been found to have the ability to downregulate CD4 receptors and major histocompatibility complex class 1 (MCH 1), which benefits HIV by interfering with viral recognition and destruction of infected cells by cytotoxic T-cells (142, 143). Nef has been shown to persist in the blood of ART-treated PWH and cause ROS generation, release of monocyte attractant protein-1 (MCP-1), and consequent apoptosis of endothelial cells (144, 145). On the other hand, these are similar processes found with the upregulation of ENaC (17) and may interact during HIV.

Both Nef and Tat have also been shown to induce NADPH oxidases (NOX) as well as immense enhancement of their responses to a variety of stimuli (146, 147). Seven distinct NOX isoforms have been identified, of which NOX1, 2, 4, and 5 have been characterized in the cardiovascular system (148). However, NOX4 is the most abundant NOX isoform in the renal tubular segments, including the ASDN, although significant amounts have also been found in the pulmonary vasculature and microglia (148–151). Animal studies have demonstrated that Tat augments NOX4, facilitating the production of ROS (151, 152). The NOX4-dependent pathway regulates many factors essential for proper sodium handling in the distal nephron (149). A study by Cowley et al. showed that Dahl salt-sensitive rats lacking NOX4 have lower blood pressure and renal injury when placed on a high salt diet than wild type controls (153). This was supported by another study by Pavlov et al. (149) which showed that NOX4 deletion in Dahl salt-sensitive rat challenged with a high salt diet attenuated high salt-induced upregulation of ENaC activity in the cortical collecting ducts. Pavlov et al. further showed that H_2_O_2_ upregulated ENaC activity, and H_2_O_2_ production was reduced in both the renal cortex and medulla in salt-sensitive rats with deleted NOX4 who were fed a high salt diet, suggesting that ENaC upregulation in the context of renal injury might result from overstimulation of the NOX4-dependent pathway and increased ROS generation (149). With evidence indicating influence of HIV proteins on NOX and generation of ROS, it can be hypothesized that HIV may have an effect on the renal ENaC salt handling and salt sensitive hypertension. However, as data is still scarce in this area, the relationship between HIV and ART on salt handling through ENaC remains unknown.

### Epithelial sodium channel and antiretroviral therapy

Metabolic risks such as weight gain, dyslipidemia and hyperglycemia, which are part of the metabolic syndrome, have been identified in patients on cART which can alter normal endothelial function (72, 134, 135, 154, 155). Sax et al. showed that weight gain was greater in more recent trials and with the use of newer integrase strand transfer inhibitor (INSTIs) such as dolutegravir than protease inhibitors (135). Sax et al. further showed that demographic factors associated with weight gain included lower CD4 cell count, higher HIV type 1 RNA, no injection drug use, female sex, and black race. Several observational studies have also indicated significant weight gain in PWH on INSTIs with some indicating a predisposition toward black race and female sex (72, 156, 157). Excessive weight gain, particularly central obesity is associated with vascular endothelial dysfunction. Healthy perivascular adipose tissue (PVAT) ensures normal endothelial vasodilation function, however, enhanced oxidative stress and pro-cytokine generation found with obesity-associated PVAT is associated with reduced NO availability promoting the aforementioned cascade of events leading to atherosclerosis (133). Obesity is also associated with elevated aldosterone and insulin levels, both of which stimulates ENaC to conserve sodium and are thought to be a likely cause of refractory hypertension in this population (51).

High levels of insulin also stimulate the renin-angiotensin-aldosterone system (RAAS) resulting in increased peripheral vascular resistance and hypertension (158). RAAS is crucial for blood pressure regulation as well as the pathogenesis of cardiovascular diseases (159, 160). The RAAS involves the secretion of renin, an enzyme secreted by the juxtaglomerular cells of the kidneys in response to various stimuli such as decreased afferent arteriolar perfusion, decreased sodium content in the macula densa and sympathetic activation (68). Renin activates the conversion of angiotensinogen to angiotensin I, which is further cleaved to into Ang II by the angiotensin converting enzyme (ACE) (68). Ang II acts on the angiotensin receptor I (AT_1_R) and induces deleterious effects such as vasoconstriction, sympathetic activation, reduction in NO synthesis, inflammation and oxidative stress; processes leading to the pathogenesis of hypertension (46, 67, 159, 160). The effects of Ang II are counteracted by another enzyme angiotensin converting enzyme II (ACE 2), which acts on the angiotensin receptor II (AT_2_R), although these receptors are more restricted in tissue expression and affinity in adults (161). The AT_2_R acts as the catalytic site for the ACE 2 which results in conversion of Ang II to angiotensin-1,7 (Ang-1,7) (161). Ang-1,7 causes vasodilatation, inhibits oxidative cellular damage by blocking NADPH-oxidase, and stimulates NO production via nitric synthase pathways (162). All of these cellular processes have a protective effect on the endothelium. However, there is limited data on whether HIV and obesity cause further downregulation of Ang-1,7 although both HIV and obesity have been shown to cause upregulation of Ang II via RAAS, potentially contributing to hypertension (73). As previously mentioned, Ang II promotes secretion of the key ENaC regulator, aldosterone, from the adrenal zona glomerulosa, which is essential for the pathogenesis of hypertension (4).

A study by Srinivasa et al. showed that HIV-infected subjects on cART with excess visceral adipose tissue had significantly higher RAAS activation than HIV-infected subjects without excess visceral adipose tissue (73). This study further showed that HIV-infected subjects with excess visceral adipose tissue had increased aldosterone and plasma renin activity compared to the HIV-infected subjects without excess visceral adipose tissue (73), suggesting the potential role of dysregulated exogenous aldosterone production and RAAS activation from adipose tissue (163, 164). In addition, aldosterone stimulates both the innate and adaptive immune systems leading to macrophages and T cells accumulation in the kidneys, heart, and vasculature which contribute to the development of hypertension and other cardiometabolic diseases (165). The persistence of immune activation in HIV (144, 166) and the metabolic toxicity associated with cART (72, 134, 135, 154, 155) may potentially play an additive or multiplicative role on the effects of aldosterone and ENaC activation, and therefore increase the risk of hypertension in PWH. Given the widespread use of dolutegravir, it is worth prospectively investigating the impact of weight gain on ENaC function in PWH.

**FIGURE 3:**
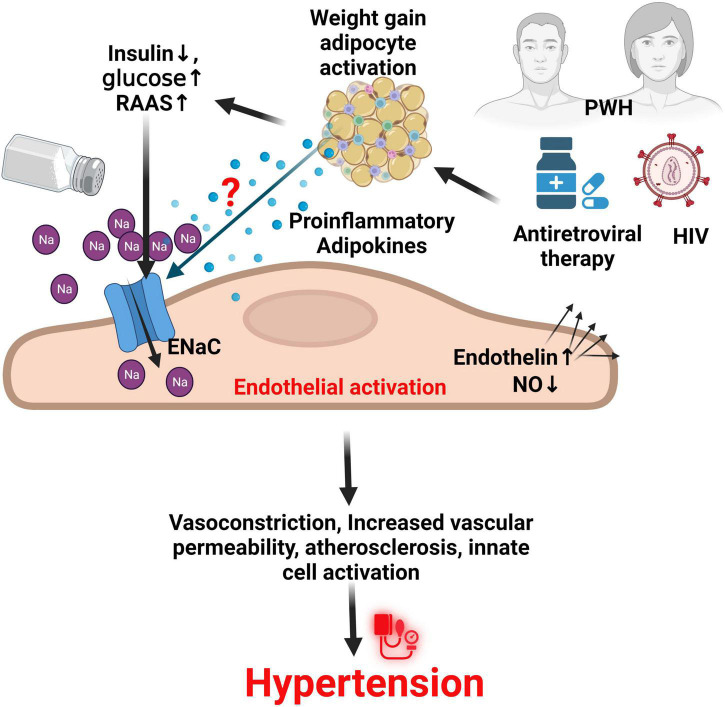
ENaC in endothelial cells and the role of HIV and ART in hypertension. It is thought that HIV infection and use of antiretroviral therapy especially dolutegravir, synergizes with other factors such as increased glucose, reduced insulin action and production, and activation of the renin angiotensin aldosterone system (RAAS) through unknown mechanisms to increase the activity of the endothelial sodium channel (ENaC) on activated endothelial cells. This results in several effects that contribute to hypertension. NO, nitric oxide; PWH, persons with HIV; Na, sodium; HIV, human immunodeficiency virus.

Several animal studies have shown that adipose cells in small arteries demonstrate increased expression of endothelin-1, endothelin type A and endothelin type B receptors, and TNF- α, which augment adrenergic vasoconstriction while impairing tonic NO release (167–170). In addition, high glucose levels lead to enhanced non-enzymatic glycation, sorbitol-myo-inositol-mediated changes, redox potential alterations, and the activation of protein kinase C which induces oxidative stress (171). The low-density lipoproteins also activate intracellular pathways to increase local and systemic inflammation, monocyte adhesion, endothelial cell dysfunction and apoptosis, and smooth muscle cell proliferation, leading to foam cell formation and genesis of atherosclerotic plaque (172, 173). The effect of these various metabolic risks occurs in a synergic fashion with HIV (27, 174–178), and also found with salt-sensitivity and ENaC activation (16, 61, 91), (Figure 3) suggesting an imperative need to investigate the contribution of Na^+^ and ENaC activity, and their interaction with anti-retroviral therapy in PWH.

## Sex differences, epithelial sodium channel and hypertension in PWH

It is worth noting that gender differences have been noted in human immunodeficiency virus (HIV) infection in different geographic areas. In 2020, women aged 15 years and older accounted for at least 56% of HIV infections in sub-Saharan Africa (SSA), while in western and central Europe and North America, the same age group accounted for only 20% (179). Although the epidemiological problem of hypertension in HIV-infected adults is well studied (65, 66, 180), gender influences on the pathogenesis of hypertension in PHW are not as well studied, despite the geographical diversity of HIV infection.

Existing studies have shown that the prevalence of hypertension is generally higher in men than women, regardless of age, although severity in women increases markedly with age (181–183). Hypertension in men is mainly attributed to low socioeconomic status and behavioral factors such as high alcohol consumption and smoking, while advancing age and hormonal imbalances from the onset of menopause and combined oral contraceptives pills (COCs) appear to be main contributors in women (181, 183). In general, women have higher CD8+ T cell activation, higher interferon-stimulated genes (which are both protective in HIV progression and accompanying comorbidities) and the protective effect of estrogen on the development of hypertension appear to be what protects women living with HIV from developing hypertension earlier than men (184). Animal studies have also shown that estrogen reduces ENaC function and blood pressure (185), providing further evidence to explain postmenopausal hypertension. More studies are therefore needed to show whether this effect extends to PWH.

Gender differences have been identified in Na^+^ storage between muscle and skin which may be one of the factors underlying sex-specific susceptibility. For example, a study by Wang et al. showed that men have higher levels of Na^+^ in their skin than in muscle, while women accumulate Na^+^ to greater levels in their muscle (186). Increased Na+ accumulation in the skin compared to muscle is associated with increased risk for the development and pathogenesis of hypertension (69, 150). Interestingly, Barbaro et al. demonstrated that monocytes from humans with high skin Na^+^ exhibited increased IsoLG-adduct accumulation and CD83 expression (91), thought to lead to immune tolerance depletion in salt-induced hypertension (62, 92). In the previous section, it was stated that soluble markers such as CD14 were found to be elevated in PWH with hypertension, with upregulation of ENaC, and with high-salt concentration (61, 89, 91). Siedner et al. found that HIV-infected women on long-term ART had significantly elevated levels of soluble CD14 and soluble CD163 compared to HIV-infected men (187). Evidence from the general population also indicates that women exhibit significant changes in blood pressure in response to high-salt consumption compared to men (188, 189). This has been attributed to factors such as failure to suppress aldosterone following high-salt load (190), a key mechanism leading to protection from salt-sensitive hypertension (191, 192). However, further studies are needed to investigate the contribution of salt-sensitivity and ENaC to hypertension along with gender differences in PWH.

There are also gender differences in factors affecting the gut microbiota in the development of hypertension. A study of over 200, 000 participants characterizing gender-specific dietary patterns showed that women were 2.4 times more likely to consume too much sugar, 1.4 times more likely to consume too much fat, and 1.4 times as likely to consume fiber intake below the recommendations (193). Excess sugar and fat, and less fiber has a tendency to tip the gut microbiota toward dysbiosis (194), which has been shown to cause increase in blood pressure in animal studies (195, 196). High dietary sodium consumption may contribute to gut dysbiosis by altering the gut microbial composition, and inducing an inflammatory response which alters the gut anatomy (197), and is associated with increased immunogenic IsoLG-adduct formation responsible for salt-sensitivity and upregulation of ENaC (61, 198), and women have been reported to be more sensitive to salt (188, 189). The group by McDonough et al. reported that in mice, sodium channels in the kidney demonstrate a sex-specific adaptation to high-salt diet that ultimately preserve electrolyte homeostasis (199). Several sodium transporters along the nephron (Na+/H+exchanger 3 (NHE3), bumetanide-sensitive Na^+^-K^+^-2Cl cotransporter (NKCC2), Na^+^-Cl^–^ cotransporter (NCC), and the ENaC) play specific roles in the ultimate maintenance of electrolyte homeostasis and changes in the activity of one may affect the expression and activity of the other transporters (200–202). ENaC particularly mediates the final step in sodium reabsorption, making it an important regulator of sodium and blood pressure (165). Sex-specific differences in the expression of sodium channels along the nephron were observed when mice were fed high salt (4% sodium chloride), but the fold changes were similar along the distal nephron and collecting duct where ENaC is located (200–202). However, compared to other transporters, inhibition of ENaC resulted in increased excretion of sodium and water in male rats compared to females suggesting that the response of ENaC in males may be more pronounced than in females (165). There is also some evidence of patho-immune gender-specific mechanisms in the development of hypertension involving IL-17 secretion, NO and Ang II (67, 203–205). A study by Zimmerman et al. showed that infusion on Ang II in Male Sprague-Dawley rats produced a greater increase in the number of renal Th17 cells (206), and Th17 cells have been associated with Ang II-induced increases in BP, salt-induced increase in SGK1 and ENaC activation (77, 96). It has also been shown that HIV promotes the production of renin by CD4 T-cells, which is a source of endogenous RAAS activation (207), however, sex differences in the HIV population need further studies.

**FIGURE 4:**
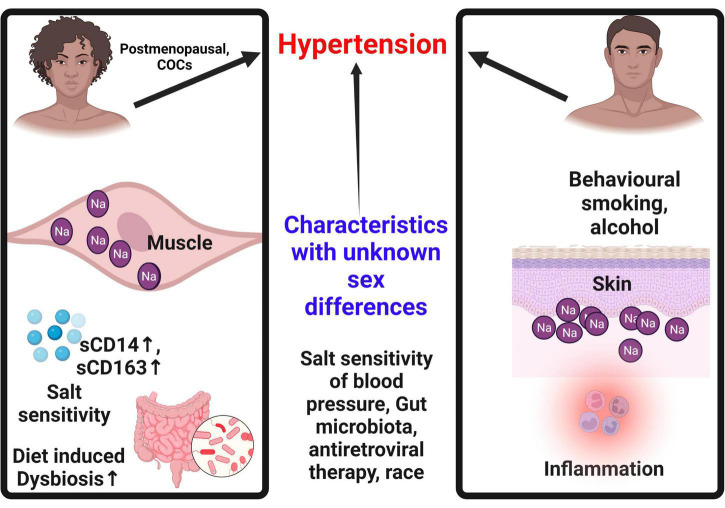
Sex differences contributing to hypertension. Sex specific characteristics contribute to hypertension. In females, hypertension is only common after menopause. Females tend to store more sodium in the muscle than in the skin, a concept that may be protective when compared to males. Males on the other hand tend to store more sodium in the skin eliciting an inflammatory cascade that contributes to hypertension. Females are more susceptible to salt sensitivity and diet induced dysbiosis that contribute to hypertension. Use of combined oral contraceptives is a predictor of hypertension in females. Some behavioral patterns common in men such as alcohol and smoking increase the risk of hypertension in males. COCs, combined oral contraceptives; Na, sodium; sCD14, soluble cluster of differentiation 14; sCD163, soluble cluster of differentiation 163.

A summary of potential sex differences contributing to hypertension are shown in Figure 4. However, a number of questions remain unanswered regarding the sexes in terms of interaction between ENaC activity, HIV infection, ROS and antiviral therapy and hypertension.

The protean external and internal gender-specific influences in the pathogenesis of hypertension, should prompt the transition from putative approaches to the development and integration of gender-specific interventions for the prevention of CVD risk factors such as hypertension in PWH. There still remain a paucity of data on the contribution of gender to the pathogenesis of hypertension and role of ENaC in HIV-infected individuals.

### Antioxidants in treatment of hypertension in human immunodeficiency virus

Evidence has indicated a favorable effect on blood pressure reduction with ENaC blockers such as amiloride, which inhibits the endothelial amiloride sensitive subunits of the ENaC, as well as aldosterone blockers such as spironolactone (208, 209). However, as the hallmark of endothelial dysfunction is driven by reduction in NO release and increase generation of ROS, which can occur in the setting of HIV, potential therapies such as antioxidant therapy can be considered in the HIV population in the control of ENaC driven hypertension. Studies have indicated that PWH, including those on cART have high levels of total plasma peroxides and oxidative stress index compared to the control group (210). Preclinical studies have demonstrated that therapies which improves NO bioavailability, such as Lazaroids (inhibitors of lipid membrane peroxidation and appear to function as oxygen free radical scavengers) have been shown to reduce blood pressure in animal models (211). Additionally, vitamin D has also been shown to reduce renin-angiotensin-aldosterone system (RAAS) activity as well as regulating vascular oxidative stress and can be considered as a potential therapy for hypertension, especially that PWH appear to have lower vitamin D- levels compared to controls (212–214). However, due to the multifaceted nature of hypertension etiology in humans, results on the use antioxidants as potential treatment for hypertension remain conflicting (215). Nonetheless, a consideration for anti-hypertensive drugs that also exert antioxidant effects such as nebivolol, carvedilol, and celiprolol is an alternative option (215). More studies are required to explore the potential for antioxidant therapy to treat hypertension.

## Conclusion

The multifactorial link between HIV and CVDs is well established. Hypertension, an important driver in the development of most CVD events, requires more robust studies. Salt-sensitive hypertension has already been demonstrated to be highly prevalent in PWH, but molecular studies such as the role of ENaC are still scarce in regions most affected by both HIV and CVDs, such as the sub-Sahara Africa. This review aimed to highlight the pathogenesis surrounding salt-sensitivity and ENaC and their contribution to the pathogenesis of hypertension in PWH, with the hope that more can be invested in preventing the onset and progression of CVDs in PWH.

## What is known?

• ENaC regulates blood pressure.
• Hypertension is more common in persons with HIV compared to persons without HIV.

## What is new?

• In HIV, the activity of ENaC is likely increased resulting in increased risk for the development of hypertension in persons with HIV.
• Some sex differences in hypertension are explained by the role of ENaC. However, the mechanisms still remain to be explored.
• Use of antiretroviral therapy likely modulates ENaC function through yet unknown mechanisms.

## Author contributions

KM conceptualized the study and wrote the draft manuscript. SM and KM wrote different sections of the manuscript. SM created all the figures. KP and NM edited and reviewed the manuscript. AK edited and reviewed the manuscript, conceptualized the framework and finalized the manuscript as well as obtained funding for the manuscript. All authors contributed to article reviews, edited and approved the final version of this manuscript.
